# Dynamic Changes in Muscle Thickness and Infrapatellar Fat Pad During Quadriceps Setting: An Ultrasound Echo Analysis

**DOI:** 10.7759/cureus.76288

**Published:** 2024-12-23

**Authors:** Takashi Hasegawa, Keita Nishi, Shinichi Nagae, Riho Tomura, Shinichi Matsumoto, Toshio Higashi

**Affiliations:** 1 Department of Rehabilitation, Wajinkai Hospital, Nagasaki, JPN; 2 Department of Health Sciences, Graduate School of Biomedical Sciences, Nagasaki University, Nagasaki, JPN; 3 Department of Physical Therapy, School of Health Science, Toyohashi Sozo University, Toyohashi, JPN; 4 Department of Rehabilitation, Furukawa Miyata Orthopedic and Internal Medicine Clinic, Nagasaki, JPN

**Keywords:** infrapatellar fat pad, isometric contraction, quadriceps setting, ultrasound echo, vastus medialis

## Abstract

Purpose

The infrapatellar fat pad (IFP) has the lowest pain threshold among all knee joint components and causes anterior knee pain after knee arthroplasty. It has been reported that selective muscle atrophy of the vastus medialis (VM) and fibrosis of the IFP may develop following knee joint surgery. Ultrasound enables visualization of IFP deformation (A1) from within the joint to the proximal area in response to muscle contraction, and this may be helpful in developing preventive and therapeutic strategies for IFP fibrosis. Therefore, this study aimed to clarify the relationship between quadriceps muscle thickness and dynamic changes in IFP during quadriceps setting (QS).

Methods

This study involved six participants (all men, 12 knees) with no history of knee joint problems, with a mean age of 36.7±8.7 years. We used ultrasound imaging to evaluate quadriceps muscle thickness and IFP dynamics. The muscle thicknesses of the VM, vastus lateralis (VL), vastus intermedius (VI) and rectus femoris (RF) were measured at rest and during QS with maximal contraction. The IFP measured the anterior-posterior width and the patellar tendon-tibia angle. The differences between conditions were examined using the Wilcoxon signed-rank test, and the correlation between the differences between measurement conditions was calculated using the Spearman rank correlation coefficient.

Results

The thickness of each muscle was measured and only the vastus lateralis muscle showed a significantly lower value during QS, while all other measurements showed higher values ​​(VM: p=0.0029, VL: p=0.0414, VI: p=0.0022, RF: p=0.0022, compared to resting). The mean anteroposterior width of the IFP increased by 0.9 mm medially (p=0.015) and 1.4 mm laterally (p=0.0076). Regarding the correlation between the measurements, a significant positive correlation was observed only between the VM difference and the lateral IFP difference (ρ=0.81, p=0.0071).

Discussion

The IFP provides a cushioning effect for the patellofemoral joint and reduces friction between the articular cartilage and the patellar ligament through functional deformation. In this study, a significant positive correlation was found between VM difference and lateral IFP difference. These findings suggest that the contractile force of the VM may be related to the extent by which the IFP is pushed outward, and changes in the flexibility of the soft tissues around the knee, such as IFP, may contribute to functional impairment in patients with knee joint disease.

Conclusion

This is the first study to quantitatively assess the extent of IFP deformation in the medial and lateral patella. The results of the study suggest that changes in soft tissue flexibility around the knee, such as in the IFP, may contribute to functional impairment in patients with knee joint disease.

## Introduction

 The infrapatellar fat pad (IFP) fills the space in front of the knee surrounded by the femur, tibia, patella, and patellar tendon [1]. Inflammation in the IFP can lead to edema, proliferation, and contractures, potentially limiting knee motion in conditions such as Hoffa syndrome and IFP contracture syndrome [2]. The IFP is rich in free nerve endings and has been reported to have the lowest pain threshold among all knee joint components, making it the key source of anterior knee pain (AKP) following knee surgery [3,4]. It has also been reported that selective muscle atrophy of the vastus medialis (VM), fibrosis of the IFP, and changes in patella height occur after knee joint surgery [5,6]. Ultrasound imaging can visualize IFP deformation from within the joint to the proximal area in response to muscle contraction, which may be useful for evaluating strategies to prevent and treat IFP fibrosis [7]. Previous studies have used magnetic resonance imaging (MRI) to measure the angle between the patellar tendon and the anterior tibia ((patellar tendon-tibial angle, (PTTA)) at each knee flexion angle and evaluate the extent of IFP deformation [8,9]. Based on these findings, when the change in the PTTA is significant, the extent of IFP deformation is significant, and the extent of IFP deformation can be predicted by measuring the PTTA. It has also been reported that during quadriceps setting (QS), the IFP spreads as if it is being pushed out to the periphery, and the thickness of the patella increases both on the inside and outside. QS is an isometric exercise aimed at strengthening the quadriceps, which is performed in long sitting position, with a rolled towel placed under the popliteal fossa. The towel is pressed down by contracting the quadriceps. Differences in the degree of quadriceps muscle contraction during QS may affect the extent of IFP deformation [10]. Given the association between IFP and AKP following knee joint surgery, verifying the effect of changes in quadriceps muscle thickness during QS, which is frequently used in postoperative rehabilitation of the knee joint on dynamic changes in IFP may aid in the treatment of patients with knee disease. Therefore, this study aimed to clarify the relationship between quadriceps muscle thickness and dynamic changes in IFP during QS.

## Materials and methods

This study included 12 knees from six men (mean age: 36.7±8.7 years), with no history of knee joint problems. Basic information about the participants was obtained, including height, weight, age, sex, and body mass index. Exclusion criteria included those with a history of orthopedic disease, cardiovascular disease or cerebrovascular disease, or those with a limited range of knee joint motion or knee pain. This study was conducted between 15 May and 14 July 2023. This study was conducted in accordance with the guidelines outlined in Declaration of Helsinki, ensuring confidentiality of personal information. Written informed consent was obtained from all participants. This study was approved by the Ethics Committee of the Wajinkai Hospital, Japan (approval number: 230502).

**Figure 1:**
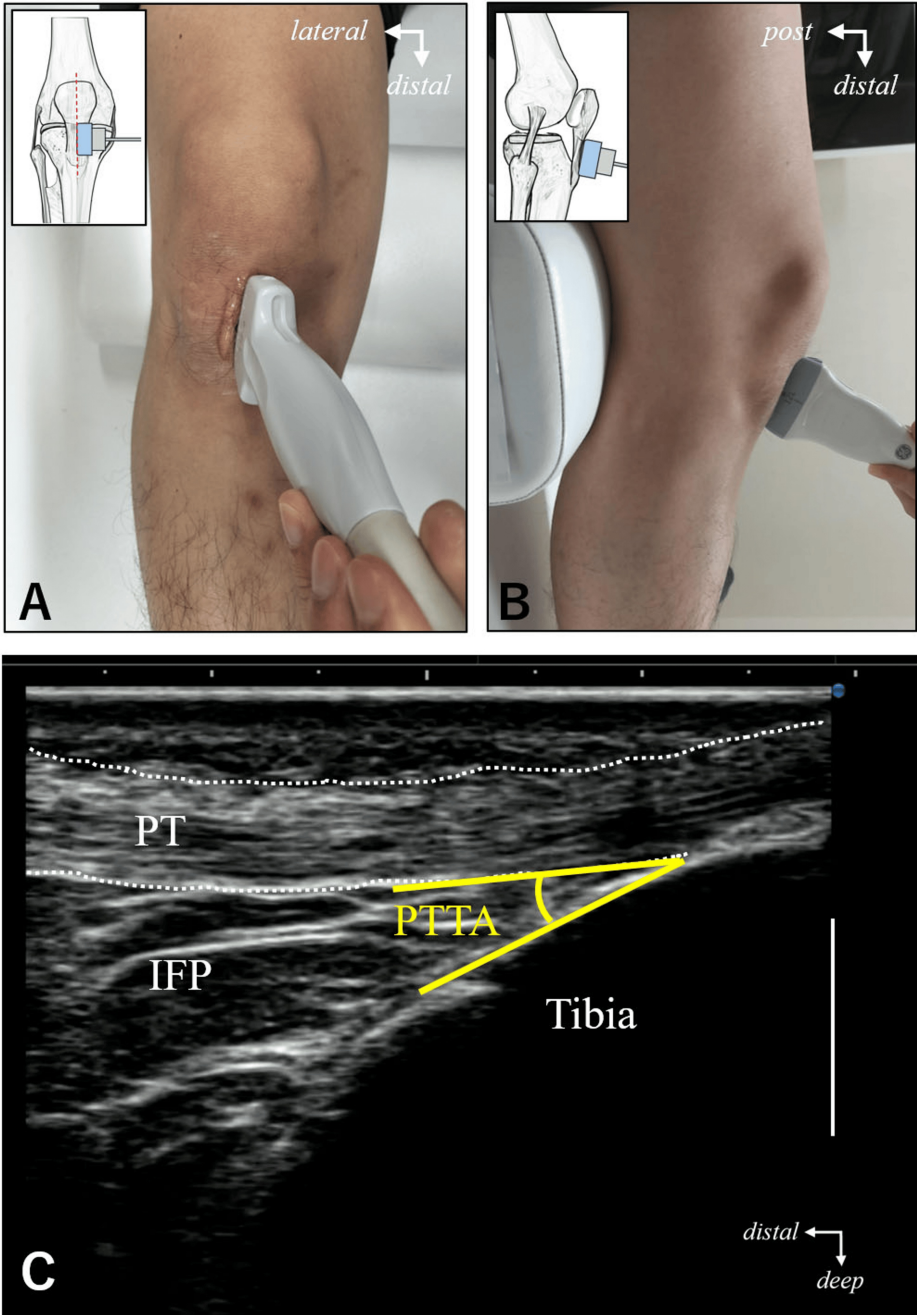
A method for ultrasound imaging of the infrapatellar fat pad at the anterior aspect of the proximal tibia. A and B are the position of the probe when measuring. A: anterior view; B: lateral view; C: ultrasound image. The angle composed of the deep border of the patellar tendon and the anterior surface of the proximal tibia was defined as the patellar tendon-tibia angle and measured. PT, patellar tendon; IFP, infrapatellar fat pad; PTTA, patellar tendon-tibia angle. Scale bar 10 mm.

 To evaluate the muscle thickness and IFP dynamics of the quadriceps femoris, we took B-mode ultrasound images using an ultrasound diagnostic device (Konica Minolta, SONIIMAGE HS2) with a 10 MHz linear probe. Furthermore, ultrasound imaging was performed by a single examiner who underwent training to ensure clear ultrasound images could be taken. The measurement position was a sitting position with the knee joint flexed at 20º. The joint angle was maintained passively using a goniometer, with a cushion placed in the popliteal fossa. We measured the muscle thickness of the VM, vastus intermedius (VI), vastus lateralis (VL), and rectus femoris (RF) at rest and during QS with maximal contraction [7]. Maximal contraction (A1) was confirmed by palpation. Difference in muscle thickness was calculated by subtracting the value at rest from the value during QS. The measurement sites for each muscle thickness were VM, 5 cm at a 45-degree angle to the line connecting the anterior superior iliac spine (ASIS) and the patella, VI and RF, at the midpoint between the ASIS and the patella, and VL, at the midpoint between the greater trochanter and lateral epicondyle [11]. The PTTA and the anteroposterior width of the distal 1/3 of the patella, medial and lateral were measured. The PTTA was measured directly above the patellar ligament, parallel to the fiber course of the patellar ligament, and a long-axis image was taken at the insertion point of the patellar ligament so that the anterior surface of the proximal tibia and IFP were visualized. The angle between the patellar tendon and the anterior surface of the proximal tibia was measured (Figure 1).

**Figure 2:**
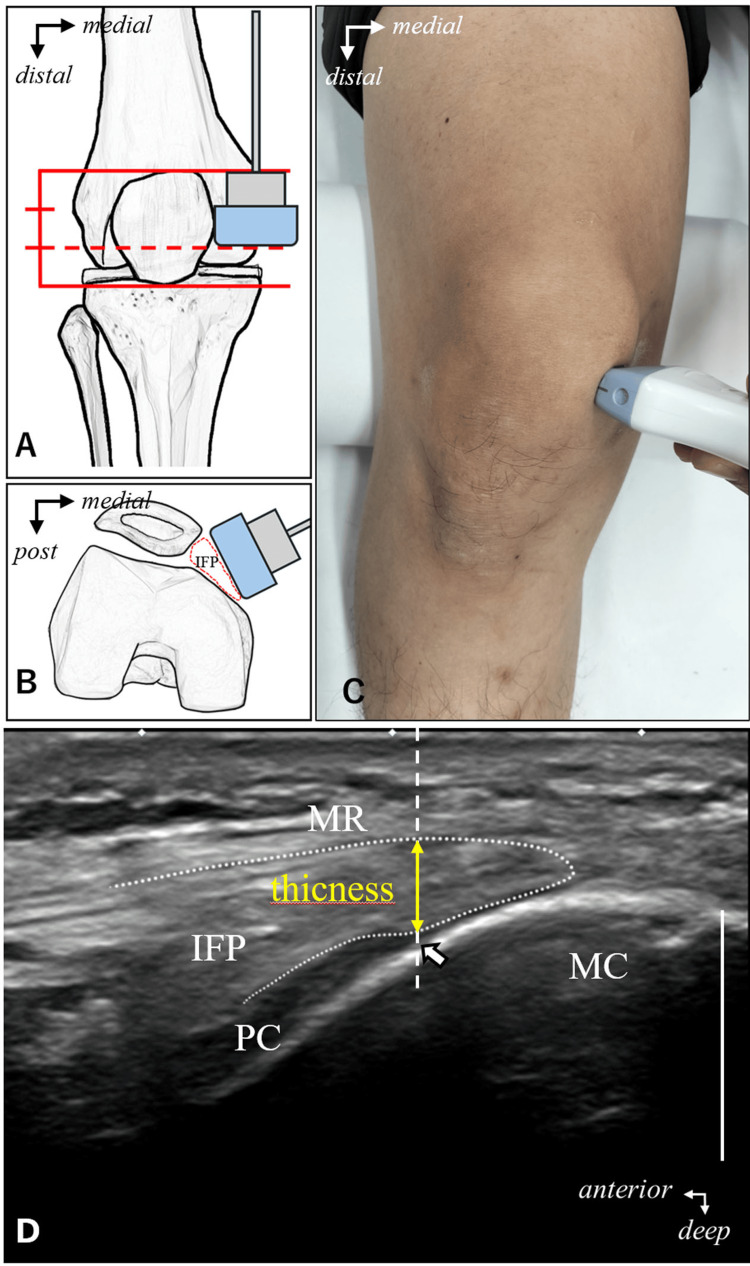
Imaging method for medial IFP thickness. A: a line was drawn medially at the distal 1/3 of the length of the long axis of the patella to determine the imaging position. B: a short-axis ultrasound image was taken with the probe placed between the patella and the tibia. C:  shows the actual imaging. The same method was used for the outer IFP thickness, and imaging was performed at the lateral part of the patella. D: shows medial IFP thickness measurement method. A vertical line was drawn at the border between the medial femoral condyle and the articular cartilage of the patellar surface, and the thickness of the IFP was measured at that point. Arrows indicate the medial edge of the articular cartilage of the patellar surface. MC: medial condyle; PC: patellar surface cartilage; MR: medial patellar retinaculum; IFP: infrapatellar fat pad. Scale bar 10 mm.

The probe was placed at the distal 1/3 level of the patella to capture the medial IFP in a short-axis image. The medial edge of the patellar surface cartilage, depicted as a hypoechoic area on the anterior medial surface of the femur, was used as the reference point, and the distance between the patellar retinaculum and the reference point was measured as the thickness of the IFP. The outside of the graft was photographed in the same way (Figure 2). The thickness of each tissue and the PTTA were calculated as the average value by three examiners.

Statistical analysis

Statistical analysis was performed using statistical analysis software (SPSS Version 22; Chicago, IL, USA), and normality was confirmed using the Shapiro-Wilk test. In addition, the differences between conditions were examined using the Wilcoxon signed-rank test, and the correlation between the differences between measurement conditions was calculated using the Spearman rank correlation coefficient. Effect sizes were interpreted based on Cohen et al.’s criteria: small (0.1≤ ρ<0.3), medium (0.3≤ρ<0.5), and large at (0.5≤ ρ)[12]. The significance level for each was set at 5%.

## Results

Changes in thickness of each tissue during rest and QS

The mean difference in muscle thickness of each quadriceps muscle between rest and QS was VM (6.1±4.6mm), VL (-1.5±2.6mm), VI (6.6±2.3mm) and RF (5.9±3.2mm) (p<0.05) (Table 1).

**Table 1:** Changes in thickness of each muscle rest and QS. Values are mean±standard deviation (SD). The statistical method used was the Wilcoxon signed-rank test, * : p<0.05 QS: quadriceps setting; VM: vastus medialis; VL: vastus lateralis; VI: vastus intermedius; RF: rectus femoris.

muscle	VM (mm)		VL (mm)		VI (mm)		RF (mm)	
Rest or QS	Rest	QS	Rest	QS	Rest	QS	Rest	QS
Thickness	25.2±3.9	31.3±4.4	13.9±4.0	20.5±4.5	18.4±3.2	24.2±3.1	24.8±2.5	23.3±3.1
Difference value （QS-Rest）	6.1±4.6 *		-1.5±2.6 *		6.6±2.3 *		5.9±3.2 *	

The mean difference in PTTA was 3.1±2.9º, the medial IFP was 0.9±1.0 mm, and the lateral IFP was 1.4±1.3 mm (p<0.05). During QS, only VL showed a reduction in thickness, whereas all other muscles demonstrated significant increases in thickness ​​(p<0.05) (Table 2).

**Table 2:** Changes in thickness of each IFP rest and QS. Values are mean ± standard deviation (SD). The statistical method used was the Wilcoxon signed-rank test,* : p<0.05. IFP: infrapatellar fat pad, QS: quadriceps setting, PTTA: patellar tendon-tibia angle.

IFP	PTTA (angle, °)		Medial IFP (thickness, mm)		Lateral IFP (thickness, mm)	
Rest or QS	Rest	QS	Rest	QS	Rest	QS
measured value	32.8±6.1	35.9±7.3	2.4±0.7	3.8±1.6	3.5±1.0	4.4±1.3
							difference value	3.1±2.9 *		0.9±1.0 *		1.4±1.3 *	
（QS-Rest）													

Correlation between differences in muscle thickness and IFP width between conditions

**Figure 3:**
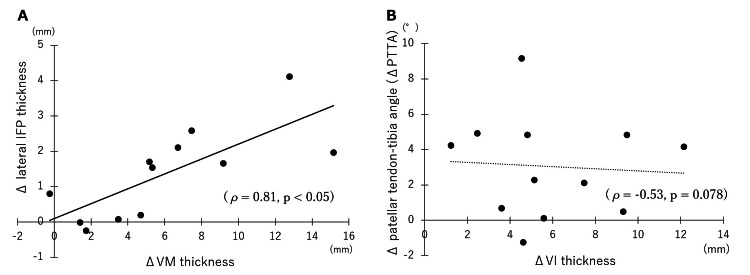
Correlation between Δ muscle thickness and Δ IFP. VM: vastus medialis; IFP: infrapatellar fat pad; VI: vastus intermedius; PTTA: patellar tendon-tibial angle.

The correlation table is shown in Table 3. A significant positive correlation was observed only between the VM difference and the lateral IFP difference (ρ=0.81, p<0.05) (Figure 3A). The change in VI muscle thickness and the change in PTTA showed a tendency toward a negative correlation (ρ=-0.53, p=0.078) (Figure 3B). No significant correlation was observed in the other measurements.

**Table 3:** Δ muscle thickness and Δ IFP correlation table. The statistical method used was the Spearman rank correlation coefficient, * : p<0.05, † : p<0.1 PTTA: patellar tendon-tibia angle; IFP: infrapatellar fat pad; VM: vastus medialis; VL: vastus lateralis; VI: vastus intermedius; RF: rectus femoris.

	ΔVM	ΔVL	ΔVI	ΔRF
ΔPTTA	0.12	-0.27	-0.53^†^	-0.25
Δ medial IFP	-0.22	0.39	0.08	-0.14
Δ lateral IFP	0.81*	-0.25	0.23	0.1

Regarding inter-examiner reliability, the intraclass correlation coefficient (1.2) for quadriceps muscle thickness and IFP during QS (rest to contraction) were VM (0.826-0.865), VL (0.804-0.866), RF (0.943-0.892), VI (0.988-0.975), medial IFP (0.803-0.822), lateral IFP (0.719-0.900), and PTTA (0.553-0.761).

## Discussion

The current study is the first to quantitatively evaluate dynamic changes in the IFP in the medial and lateral patella. Many recent studies have evaluated fibrosis and associated elastic changes in the IFP via ultrasound [4,13,14]. Advances in ultrasound elastography technology have made it possible to assess the elasticity of soft tissues, and have been applied in evaluation of the IFP [4,13]. There have also been reports of the use of dynamic ultrasonography to evaluate morphological changes in the IFP during exercise [14]. Ultrasound is simpler, more cost-effective, noninvasive, and safer than MRI, but the assessment techniques performed in these aforementioned studies are expensive additional and optional features of ultrasound, and cannot be performed in many clinical centers. Conversely, the evaluation method used in the present study can be performed in many clinical facilities because it uses B-mode, a standard feature of ultrasound imaging. If this method for evaluating dynamic changes in the IFP can be established via future validation in actual patients, it could be generalized to many clinical settings and provide useful insights into the IFP.

Changes in thickness of each muscle during rest and QS

In this study, we used ultrasound echo to examine the changes in muscle thickness, medial and lateral anterior width of the IFP, and PTTA of each quadriceps muscle during QS in healthy adult men. Muscle thickness significantly increased in the VM, VI, and RF during QS, and was significantly decreased in the VL. In the IFP, medial and lateral anterior width and PTTA significantly increased after QS. The results of the change in quadriceps muscle thickness during QS were similar to those reported by Kawai [15]. The decrease in VL muscle thickness reflects its unique contraction pattern, where thickness shifts outward and forward upon contraction. We believe that the change in the shape of the vastus lateralis on the horizontal plane associated with muscle contraction was the cause of the decrease in muscle thickness. Furthermore, the decrease in VL thickness may be due to the measurement site of this muscle. Although ultrasound is considered to be unsuitable for measuring the thickness of deep muscles such as the VI, its effectiveness in measuring superficial muscles including the VL has been well established, so we believe that it is necessary to verify the cause of this in the future [16].

Changes in thickness of each IFP during rest and QS

The extent of IFP deformation was measured by defining the lower part of the patella as the PTTA and the IFP on the medial and lateral sides of the patella as the border between the femoral condyle and the medial and lateral patellar retinaculum in the short-axis image. Both the PTTA and the medial and lateral IFP showed a significant increase after QS. A study examining the dynamics of the IFP using the ultrasound echo reported that quadriceps contraction straightens the concave patellar tendon and moves the IFP forward, filling the space between the patellar tendon and the anterior surface of the tibial tuberosity [17]. In this study, the PTTA, which indicates the extent of anterior movement of the inferior part of the IFP, also increased with quadriceps muscle contraction, supporting previous studies. A new finding in this study was the positive correlation between the increase in VM muscle thickness during QS and the increase in the anterior-posterior width of the lateral side of the IFP. There have been no previous studies showing such results, and we speculated that contraction of the VM would cause the patella to move medially, narrowing the medial sub-patellar space, pushing the medial IFP outward, and increasing the thickness of the lateral IFP. In recent years, the presence of a proximal IFP located on the medial and lateral sides of the patella has been reported. In a report examining 36 knees from fresh cadavers, 100% had a superomedial type IFP, 83% had a superomedial type IFP, and 11% had a loop type IFP [18]. The IFP is a dynamic structure that, together with the synovial folds of the knee joint, provides internal support for the patella and influences external support for the patellar retinaculum [19]. These findings suggest that the IFP is affected by patellar movement and patellar retinaculum tension via quadriceps muscle contraction. It has been reported that isometric contraction of the quadriceps moves the patella proximally, and the patellar tendon compresses the infrapatellar region, including the IFP, causing it to protrude to both sides of the patellar tendon. In addition, a study using Biodex reported that selective contraction of the VM increases the medial movement and tilt of the patella [20]. It has also been reported that in healthy knees, the surrounding knee tissues, especially the lateral patellar retinaculum, have the flexibility to accommodate the movement of the IFP, suggesting that the contractile force of the VM may be related to the extent by which the IFP is pushed outward. However, in this study, we were unable to measure patellar alignment using ultrasound echo, as was done by Asayama et al., nor could we evaluate soft tissue tension. Consequently, the effect of muscle contraction on the tissues around the knee remains unclear [21]. In addition, the extent of change in the anterior-posterior width of the IFP in this study may have been lower than that reported in previous studies, both internally and externally. Furthermore, no correlation was observed in this study between the extent of movement of the IFP internally and externally. Given that the volume and dynamics of IFP may be influenced by the participant characteristics, we intend to increase the sample size and conduct further verification in future studies. In this study, no correlation was found between VM contraction and medial IFP thickness. However, we believe that by evaluating muscle and IFP thickness during QS, we can observe dynamic changes in IFP and clarify the relationship between muscle strength and IFP.

Limitations of this study

The first limitation of this study was the insufficient inter-examiner reliability of echography. Specifically, the PTTA showed low reliability. Previous studies have reported that the PTTA changes significantly with load, and muscle contraction may also change the PTTA [8]. In this study, we attempted to standardize the measurements by placing a cushion in the popliteal fossa; however, it is necessary to reconsider positioning during ultrasound irradiation. The second limitation is that the sample size is small. In this study, a correlation was observed between VM muscle thickness and the extent of change in the lateral IFP; however, some items showed a tendency toward correlation. Therefore, it is necessary to increase the sample size and verify this. In addition, this study only targeted men, and it is possible that the relationship between sex, BMI and IFP volume also affects the amount of change in the measurements [22]. In the future, we would like to examine the relationship between dynamic changes in muscle tissue, IFP and knee function (pain and walking performance) in patients with knee disease.

## Conclusions

This is the first study to quantitatively assess the extent of IFP deformation in the medial and lateral patella. Regarding the correlation between the measurements, a significant positive correlation was observed only between the VM difference and the lateral IFP difference. These results suggest that changes in the flexibility of the soft tissues around the knee, such as IFP, may contribute to functional impairment in patients with knee joint disease. The evaluation methods described herein have the potential to be widely generalized in clinical practice, and may contribute to a better understanding of pathophysiology related to the IFP.
